# UTF1, a Putative Marker for Spermatogonial Stem Cells in Stallions

**DOI:** 10.1371/journal.pone.0108825

**Published:** 2014-10-01

**Authors:** Heejun Jung, Janet F. Roser, Minjung Yoon

**Affiliations:** 1 Department of Animal and Biotechnology Science, Kyungpook National University, Sangju, Korea; 2 Department of Horse, Companion and Wild Animal Science, Kyungpook National University, Sangju, Korea; 3 Department of Animal Science, University of California Davis, Davis, California, United States of America; Ecole Normale Superieure de Lyon, France

## Abstract

Spermatogonial stem cells (SSCs) continuously undergo self-renewal and differentiation to sustain spermatogenesis throughout adulthood in males. In stallions, SSCs may be used for the production of progeny from geldings after cryopreservation and therapy for infertile and subfertile stallions. Undifferentiated cell transcription factor 1 (UTF1) is a putative marker for undifferentiated spermatogonia in humans and rats. The main purposes of this study are to determine the following: 1) changes in the expression pattern of UTF1 at various reproductive stages of stallions, 2) subpopulations of spermatogonia that express UTF1. Testicular samples were collected and categorized based on the age of the horses as follows: pre-pubertal (<1 yr), pubertal (1–1.5 yr), post-pubertal (2–3 yr), and adult (4–8 yr). Western blot analysis was utilized to determine the cross-activity of the UTF1 antibody to horse testes tissues. Immunohistochemistry was conducted to investigate the UTF1 expression pattern in germ cells at different reproductive stages. Whole mount staining was applied to determine the subpopulation of UTF1-positive spermatogonia. Immunohistological analysis showed that most germ cells in the pre-pubertal and pubertal stages were immunolabeled with UTF1, whereas only a few germ cells in the basal compartment of the seminiferous tubule cross-sections of post-pubertal and adult tissues were UTF1-positive. No staining was observed in the Sertoli or Leydig cells at any reproductive stages. Whole mount staining showed that A_s_, A_pr_, and chains of 4, 8, 16 A_al_ spermatogonia were immunolabeled with UTF1 in the post-pubertal stallion tubule. Isolated single germ cells were also immunolabeled with UTF1. In conclusion, UTF1 is expressed in undifferentiated spermatogonia, and its antibody can be used as a putative marker for SSCs in stallions.

## Introduction

Spermatogonial stem cells (SSCs) undergo self-renewal and differentiation for continuous sperm production throughout adult life. It has become increasingly evident in mice that A_single_ (A_s_), A_paired_ (A_pr_), and A_aligned_ (A_al_) spermatogonia are undifferentiated spermatogonia [1], [2], which later express the c-kit tyrosine receptor as a commitment step to undergo differentiation into spermatozoa [2]. In stallions, 8 subtypes of spermatogonia, including A_s_, A_pr_, A_al_, A_1_, A_2_, A_3_, B_1_, and B_2_ spermatogonia were recently introduced [3]. The subpopulation of undifferentiated spermatogonia in stallions has not been established, although A_s_, A_pr_, and A_al_ spermatogonia are considered candidates for SSCs [3], [4].

Due to the absence of a reliable marker for SSCs, information on SSCs had been limited until Brinster and coworkers developed a technique for SSC transplantation into the seminiferous tubules of infertile recipient mice in 1994 [5]. Since then, monitoring germ cell colony formation in the seminiferous tubules of recipient mice after transplantation has been used as the key marker for monitoring the presence and activity of SSCs. The application of this SSC transplantation technique to stallions has also been introduced [6]. Transplantation of stallion testicular germ cells in immunocompromised infertile recipient mice resulted in spermatogonial colony formation; however, spermatogenesis was arrested at the spermatogonial stage due to the incompatibility of donor germ cells to the testicular environment of the recipient [6]. Highly conserved molecules expressed in SSCs are also powerful tools for studying undifferentiated spermatogonia. The putative SSC markers have been used in the localization of SSCs in fixed testicular tissues and SSC colonies of seminiferous tubules using immunohistochemical and whole mount staining techniques, respectively [2], [7]. Three molecular markers, GFRα1, PLZF, and CSF1R, have been identified as markers for undifferentiated spermatogonia in stallion, donkeys, and mules [3]. However, expression pattern of these molecules at early reproductive stage, the precise size of spermatogonia colony expressing these molecules by whole mount staining, and possible use of these molecules for immunocytochemistry have remained unclear.

Undifferentiated embryonic cell transcription factor 1 (UTF1) is a molecule expressed in embryonic stem cells [8]. UTF1 plays a key role in embryonic carcinoma and ES cell differentiation [8]. UTF1 is also expressed in spermatogonia throughout the human male gonadal development and adult testes [9]. In rats, UTF1 expression is evident in all gonocytes in embryonic and neonatal testes, although restricted to the small subpopulation of the spermatogonia A during testicular development [10]. Previous studies have suggested that UTF1 is a conserved molecule of undifferentiated spermatogonia and might play critical roles in the self-renewal of SSCs in mammals [9], [10]. The main purposes of this study are: 1) to determine the changes in the expression pattern of UTF1 at various reproductive stages in stallions, 2) to identify subpopulations of spermatogonia that express UTF1. Based on previous findings on other species, we hypothesize that UTF1 is expressed in a subpopulation of undifferentiated spermatogonia in post-pubertal and adult stallions, and UTF1 is a putative marker for stallion SSCs. The results of this study and the developed germ cell isolation procedure could be applied to unveil biological activity of SSCs, and it could also be possibly used in infertile and subfertile stallion therapy.

## Materials and Methods

### 1. Animals

Testicular samples were collected through a routine field castration service at private horse farms in the Republic of Korea and through routine castration procedures performed at University of California Davis Veterinary Medical Teaching Hospital. The testes were collected from light-horse breeds including Thoroughbred, Quarter, and Jeju horses. The reproductive stages of the horses were categorized based on the age of horses as follows: pre-pubertal (<1 yr), pubertal (1–1.5 yr), post-pubertal (2–3 yr), and adult (4–8 yr) [11]. Testes were collected during a routine castration service, but not for this study. Because the colts or stallions were not valuable to be a stud, owners of these horses made a decision for the castration to get rid of stallion like behaviors and wanted to use them for a general riding purpose. The colts and stallions were brought in to the UC Davis Veterinary Medical Teaching Hospital by owner. The castration procedure was not modified for this research. Therefore, no approval by an Institutional Animal Care and Use Committee (IACUC) or equivalent animal ethics committee was required for this research. The same testicular samples were also used for several previous studies [11]–[14].

### 2. Testicular tissue sample preparation

Testicular tissue samples were prepared as previously described [11], with minor modifications. Briefly, testes were collected and transported to the laboratory in a 4°C icebox. For fixation, testicular parenchyma (1 cm^3^) was immersed in 4% paraformaldehyde for at least 24 h. Following a dehydration procedure using series of ethanol concentrations, the tissues were embedded in paraffin. Pieces of testicular tissue (0.5 cm^3^) were removed and snap-frozen in liquid nitrogen. Frozen tissues were stored at −80°C for western blot analysis.

### 3. Western blot analysis

Western blot analysis was conducted to verify the specificity of human anti-UTF1 antibody to equine UTF1 using a previously reported protocol [11] with minor modifications. Briefly, small piece of stallion testicular tissue were removed, snap-frozen in liquid nitrogen, and stored in a deep freezer (–80°C). For protein sample preparation, frozen samples were thawed out in a 38°C water bath and then homogenized using a Polytron PT 1200 CL (Kinematica AG, Littau-Lucerne, Switzerland) in a radioimmunoprecipitation assay buffer solution for about 5 min. The protein concentration of each sample was measured using a Bradford Bio-Rad Total Protein assay (Bio-Rad Laboratories, Inc., Hercules, CA). The sample preparation buffer [0.5 M Tris-HCL (pH 6.8), 0.1% glycerol (w/v), 10% sodium dodecyl sulfate (SDS; w/v), 0.05% 2-β-mercaptoethanol (w/v), and bromophenol blue in distilled water] was used to dilute the homogenized tissues to a concentration of 1 mg/mL. After heating the samples in a boiling water bath for 15 min, samples (15 µL) were loaded into a 10% SDS-polyacrylamide gel and separated by a Mini-Protean II system (Bio-Rad). Proteins were electro-transferred to a membrane (Millipore, Bedford, MA, USA) and blocked with a Blotto reagent (Santa Cruz Biotechnology). The membrane was then incubated with the UTF1 antibody (Millipore, Billerica, MA, USA, AB3383, 1∶500) diluted in the Blotto reagent overnight at 4°C. For the negative control, the membrane was treated with normal rabbit serum (Sigma) using the same immunoglobulin (IgG) concentration as that of the primary antibodies. For the secondary antibody, anti-rabbit IgG HRP (Cell Signaling, Boston, MA) was used at a 1∶10,000 dilution for 1 h at room temperature. For film exposure, DEVELOPER (1∶10, ILFORD, Cheshire, England) and HYPAM solution (1∶5, ILFORD) were used to develop the film.

### 4. Immunohistochemistry

The immunohistochemical staining (colorimetric) of the UTF1 molecule was performed as previously described [11], with minor modifications. Tissues from pre-pubertal (n = 3), pubertal (n = 3), post-pubertal (n = 3), and adult (n = 3) stallions were used. Briefly, paraffin wax on the slides was removed by treatment with xylene (Fisherbrand, Hampton, New Hampshire, USA), and tissues were dehydrated in a series of 100%, 90%, and 70% ethanol baths. After antigen retrieval in citrate buffer at 95°C for 30 min, the tissues were treated with an unmasking solution (Vector Laboratories, Burlingame, CA, USA). The slides were immersed in 0.3% hydrogen peroxide in methanol (Fisher Scientific, Pittsburg, PA, USA) to quench any endogenous hydrogen peroxidase present in the tissues. After blocking with normal goat serum (in Vectastain Elite ABC kit, Vector Laboratories, USA) for 30 min, the tissues were treated with the UTF1 antibody (Millipore) at a dilution of 1∶500 overnight. For negative controls, sections were incubated with normal rabbit serum (Sigma, St. Louis, MO, USA) using the same IgG concentration as that of the primary antibody. After secondary antibody incubation using goat anti-rabbit biotinylated antibody (Vectastain Elite ABC kit, Vector Laboratories, USA), tissue samples were then incubated with an avidin–biotin horseradish peroxidase complex (ABC reagent) for 45 min. A Vector AEC peroxidase substrate kit was used as substrate. Counterstaining was accomplished by briefly dipping the slides in hematoxylin (ImmunoMaster Hematoxylin; American MasterTech Scientific, Inc., Lodi, CA, USA) and tissues were mounted onto glass slides with Faramount™ aqueous mounting medium (Dako, Glostrup, Denmark).

### 5. Immunofluorescence

Testicular tissues from pre-pubertal (n = 3), pubertal (n = 4), post-pubertal (n = 6), and adult (n = 6) stallions were used for immunofluorescence as previously described [15]. Briefly, 5-µm sections of testicular tissues were treated with xylene to remove the paraffin and then rehydrated in a graded series of ethanol washes. The tissues were incubated in citrate buffer at 95°C for 30 min for antigen retrieval and blocked with 5% donkey serum (Sigma, St. Louis, MO, USA) diluted in phosphate-buffered saline (PBS). The rabbit anti-human UTF1 antibody was diluted at a ratio of 1∶500 in blocking buffer and allowed to cross-react with the slides for 1.5 h in a humid chamber. The primary antibody was detected using donkey anti-rabbit IgG Alexa Fluor 488 (1∶1,000 dilution, Life Technologies, Grand Island, NY, USA). Tissues were mounted with Vectashield mounting medium containing 4,6-diamidino-2-phenylindole (DAPI, Vector Laboratories, Burlingame, CA, USA). Goat anti-human GATA4 antibody (1∶200) and goat anti-human deleted in azoospermia-like (DAZL) antibody (1∶200, Sigma, USA) were used as counterstain. Donkey anti-goat IgG Alexa Fluor 594 (1∶1000, Life Technologies, Grand Island, NY, USA) was used as a secondary antibody for these counterstains. Tissues were mounted in Vectashield mounting medium containing DAPI (Vector Laboratories).

For immunocytochemistry, single germ cells were isolated from testes in pubertal (n = 3) and post-pubertal stage (n = 3) stallions using a two-step enzyme protocol as previously reported [15], with slight modifications. Briefly, a chunk of tissue (10 g) was removed from each testis and sliced (1 cm^3^). For initial enzymatic digestion, the tissues were incubated with collagenase type IV (1 mg/mL; Sigma) dissolved in Hank’s balanced salt solution (HBSS; Invitrogen) for 10–15 min with vigorous shaking in a 37°C shaking incubator (Vision Scientific, Yuseong Gu, Daejeon, Korea). Dispersed seminiferous tubules were pelleted by centrifugation at 200×*g* and the supernatant that contained Leydig cells was removed. The tubules were then digested with trypsin (2.0 mg/mL trypsin plus 1.04 mM EDTA; Invitrogen) and DNase I (1.4 mg/mL; Sigma) in HBSS for 15 min, and the digestion was quenched using fetal bovine serum (FBS, 10%). The testicular cell solution was filtered through a 70-µm Cell Strainer (Becton Dickinson and Company, Franklin Lakes, NJ, USA). After centrifugation at 600×*g* for 10 min, the pellets were resuspended in minimum essential medium α (MEMα) supplemented with 10% FBS. Approximately 5×10^4^ germ cells in MEMα supplemented with 10% FBS were loaded onto Fisherbrand™ Superfrost/Plus microscope slides (Fisher Scientific, Fisher Scientific Company, Ottawa, Canada) and subjected to ice-cold methanol as fixation. After overnight air-drying, cells were blocked with donkey serum and followed procedure listed above for immunofluorescence.

### 6. Immunofluorescent staining of whole-mount tubules

During the germ cell separation process, the dispersed seminiferous tubules were collected and fixed in 4% paraformaldehyde overnight at 4°C. After washing with PBS for 3 times at 60-min intervals, tubules were dehydrated in a series of 25%, 50%, 75%, 95%, and 100% methanol (MeOH) for 10 min and permeabilized in 3 mL of MeOH:DMSO:H_2_O_2_ (4∶1∶1) for 3 h. The tubules were re-hydrated in 3 mL of 50% and 25% MeOH in PBS for 10 min and washed in PBS (2 times, 15 min each). The tubules were blocked with 3 mL of ice-cold PBSMT blocking buffer (2% Blotto milk powder and 0.5% Triton X-100 in PBS) for 2×15 min and 1×2 h and incubated with the UTF1 antibody (1∶500, UTF1; Millipore) diluted in blocking buffer at 4°C overnight. After washing with PBSMT (2×15 min, 5×1 h), the tubules were reacted with donkey anti-rabbit IgG Alexa Fluor 488 (1∶1,000, Life Technologies) diluted in blocking buffer at 4°C overnight. After washing with ice-cold PBSMT (2×15 min, 5×1 h) followed by PBS (2×10 min), the tubules were mounted on the Fisherbrand™ Superfrost/Plus microscope slides (Fisher Scientific, Fisher Scientific Company) with Vectashield mounting medium containing DAPI (Vector Laboratories).

### 7. Imaging

The immunostained tissues were examined using a Leica DM 2500 fluorescent microscope (Wetzlar, Germany) equipped with an EL 6000 external light source (Leica, Wetzlar, Germany), and images were captured using Leica DFC 450 C camera. Green and red fluorescent signals were observed using a dual-emission FITC/TRITC filter. The immunolabeling of single germ cells was observed using a confocal laser scanning microscope (Carl Zeiss, LSM 700). Images were captured using a LSM T-PMT camera (Carl Zeiss, LSM 700). Cell counting was performed manually by a well-trained observer. Cells stained with green or red fluorescent were considered positive cells, whereas cells not stained with any color of fluorescent were considered negative cells.

### 8. Statistical analysis

The UTF1-positive cell populations in the stallion seminiferous tubule cross-sections at post-pubertal and adult stage were statistically analyzed using a *t*-test analysis (Excel, Microsoft 2007). Approximately 100 microscopic fields per testicular tissue section were examined. Differences in the ratio of UTF1 positive spermatogonia at various reproductive stages were statistically analyzed by one way analysis of variance [ANOVA, PROC GLM: SAS version 9.0 (SAS Institute, Cary, NC)] followed by Tukey’s post-hoc comparisons. The ratio of UTF1 and/or DAZL positive spermatogonia at each reproductive stage was obtained by counting approximately 2000 spermatogonia in each testis (n = 3 in each reproductive stage). P-values<0.05 were considered statistically significant.

## Results

### 1. Cross-activity of UTF1 antibody in horse testes

**Figure 1:**
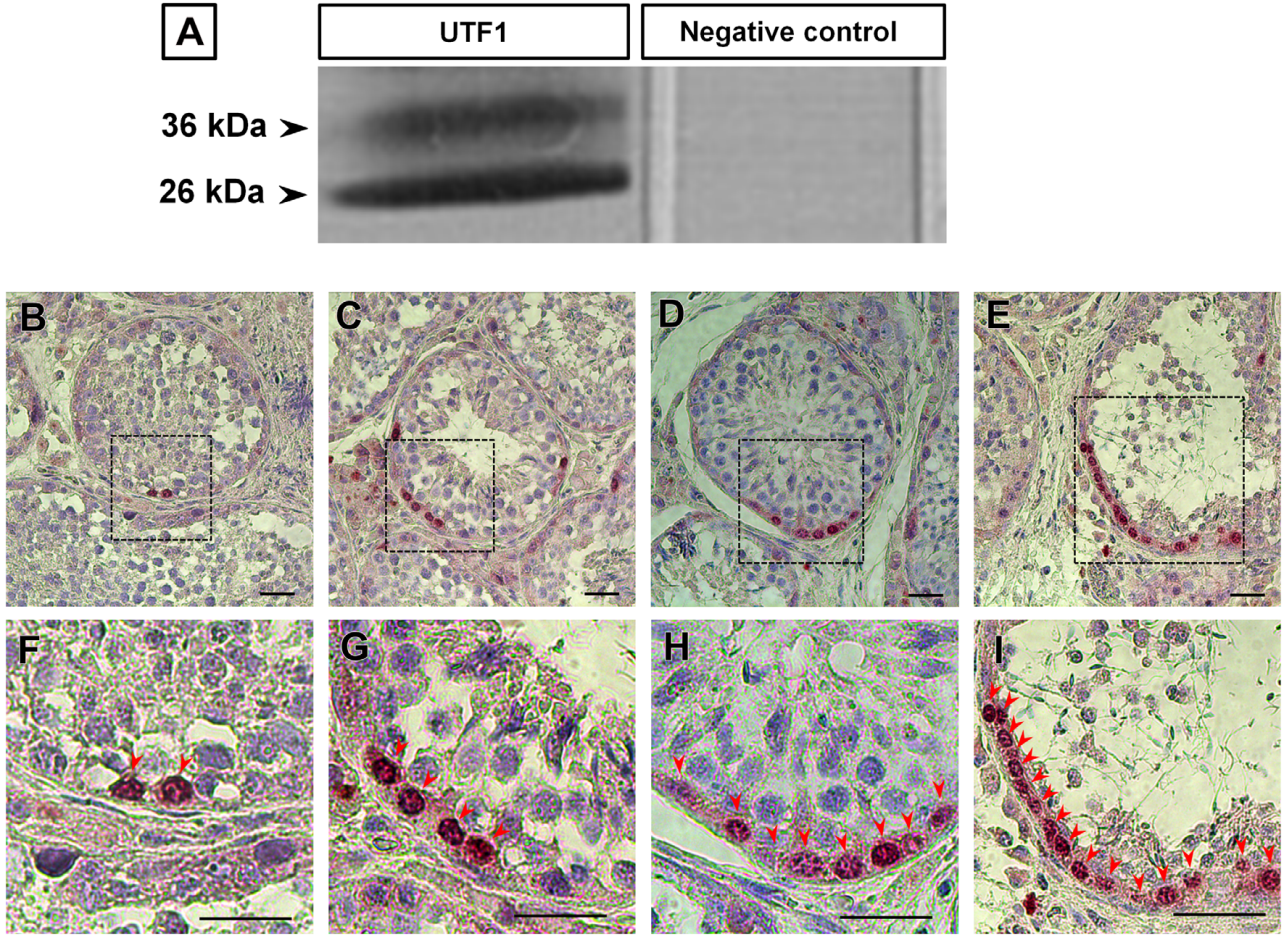
Cross-activity of UTF1 antibody in horse testes and various patterns of UTF1-positive germ cells in the seminiferous tubule cross-section. A protein band was observed at a molecular weight between 26 and 36 kDa. The negative control lane treated with rabbit serum using the same IgG concentration as that for the primary antibody showed no bands (A). Various population of UTF1 positive germ cells were observed in the seminiferous tubule cross-section of post-pubertal and adult testicular tissues. The area indicated by the broken line-black box (e.g., B, C, D and E) is enlarged (e.g., F, G, H and I). The red arrow head indicates UTF1-positive germ cells. Bar = 200 µm.

To verify the cross-activity of the UTF1 antibody to horse testes, western blot was performed using the testicular tissue extract from post-pubertal and adult stallions. The protein band showed an approximate molecular weight between 26 and 36 kDa [8]; on the other hand, no band was present on the negative control lane, which was treated with rabbit serum using the same IgG concentration as that of the primary antibody (Fig. 1A). This result confirms that the UTF1 antibody used in the present study cross-reacted with the UTF1 molecules expressed in the spermatogonia of stallion testes.

### 2. Stage-dependent immunolocalization of UTF1 in stallion germ cells

**Figure 2:**
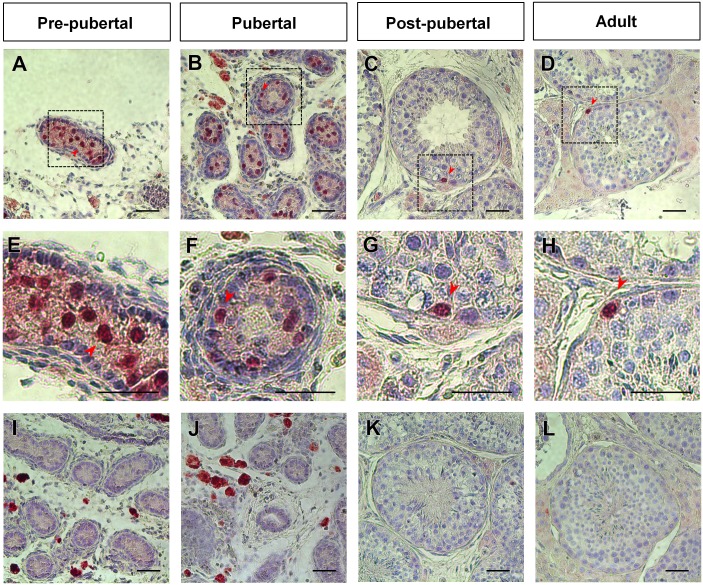
Stage-dependent UTF1-positive germ cells in stallion testis. At the pre-pubertal stage, germ cells located within the seminiferous tubules were immunostained with UTF1 (A and E). At the pubertal stage, lumen opening and incomplete spermatogenesis were evident in the seminiferous tubules (B and F). At this stage, some UTF1-positive spermatogonia were attached to the basement membrane, whereas others were located in close proximity to the tubule membrane (B and F). At the post-pubertal and adult stages, spermatogonia that resided basally within seminiferous tubules were stained with UTF1 (C, D, G, and H). The shape of UTF1-positive germ cell membrane oriented toward the lumen of the tubules was round, whereas the membranes resting on the basal lamina were flattened (C, D, G, and H). UTF1 immunolabeling was not observed in Leydig, Sertoli, or myoid cells at any reproductive stages (A–H). Negative controls treated with normal goat serum instead of primary antibody show no immunolabeling (I, J, K, and L). Lipofuscin is present in pre-pubertal and pubertal stage testes (A, B, F, I, and J). The broken lines and black boxes (e.g., A, B, C, D) were enlarged in the lower panel (e.g., E, F, G, and H). The red arrow head indicates round spermatids. Bar = 200 µm.

Immunohistochemistry (colorimetric) was performed to investigate the morphological characteristics and localization of UTF1-positive germ cells within tubules. Various UTF1 positive spermatogonia population was found in the seminiferous tubule cross-sections (Figs. 1 B–I) in adult testes. The UTF1 staining pattern in testicular tissues was investigated at various reproductive stages (pre-pubertal, pubertal, post-pubertal, and adult). At the pre-pubertal stage, spermatogonia were located distal from the basal compartment of the seminiferous tubules, and most of them were immunostained with UTF1 (Figs. 2A and E). Within the spermatogonia, UTF1 expression was exclusively observed in the nuclei of the spermatogonia (Figs. 2A and E). At the pubertal stage, the lumen opening and incomplete spermatogenesis were observed in the seminiferous tubules (Figs. 2B and F). At this stage, immunostaining was observed in the round-shaped nuclei of the spermatogonia (Figs. 2B and F). Interestingly, some UTF1-positive spermatogonia were directly attached to the basement membrane, whereas others were not attached to the membrane (Figs. 2B and F). It appears that as stallions mature from pre-pubertal to pubertal, germ cells are translocated from the luminal compartment to the basal compartment of the tubules. At the post-pubertal and adult stages, the UTF1 protein was also observed in the spermatogonia, and the UTF1-positive cells resided basally within the seminiferous tubules (Figs. 2C, D, G, and H). The nuclear shapes of the UTF1-positive cells ranged from slightly oval to round. The shape of UTF1-positive germ cell translocating from the membrane and moving toward the lumen of the tubules was round, whereas the membrane resting on the basal lamina was flattened (Figs. 2C, D, G, and H). UTF1-positive germ cells were rarely observed in the round tubule cross-sections. The mean of UTF1-positive germ cell population on the round cross-section of 100 seminiferous tubules was 1.28±0.41 (n = 3) and 1.59±0.29 (n = 3) at post-pubertal and adult stages, respectively. The UTF1-positive cell population per tubule cross-section was not significantly different between post-pubertal and adult horses (*p*>0.05). UTF1 immunolabeling was not observed in the interstitial space where Leydig cells are localized at various reproductive stages (Figs. 2A–H). At the pre-pubertal and pubertal stages, an intense fluorescent signal was observed in the interstitial space of tissues reacted with the UTF1 antibody (Figs. 2A, B, and F), as well as in the tissues treated with rabbit serum (Figs. 2I and J, negative control). This non-specific staining appears to be due to lipofuscin, a pigment granule composed of lipid-containing residues of lysosomal digestion [16]. This non-specific staining was previously reported in stallion testis of the same reproductive stages [11].

### 3. Clonal organization of UTF1-positive spermatogonia in the seminiferous tubules

**Figure 3:**
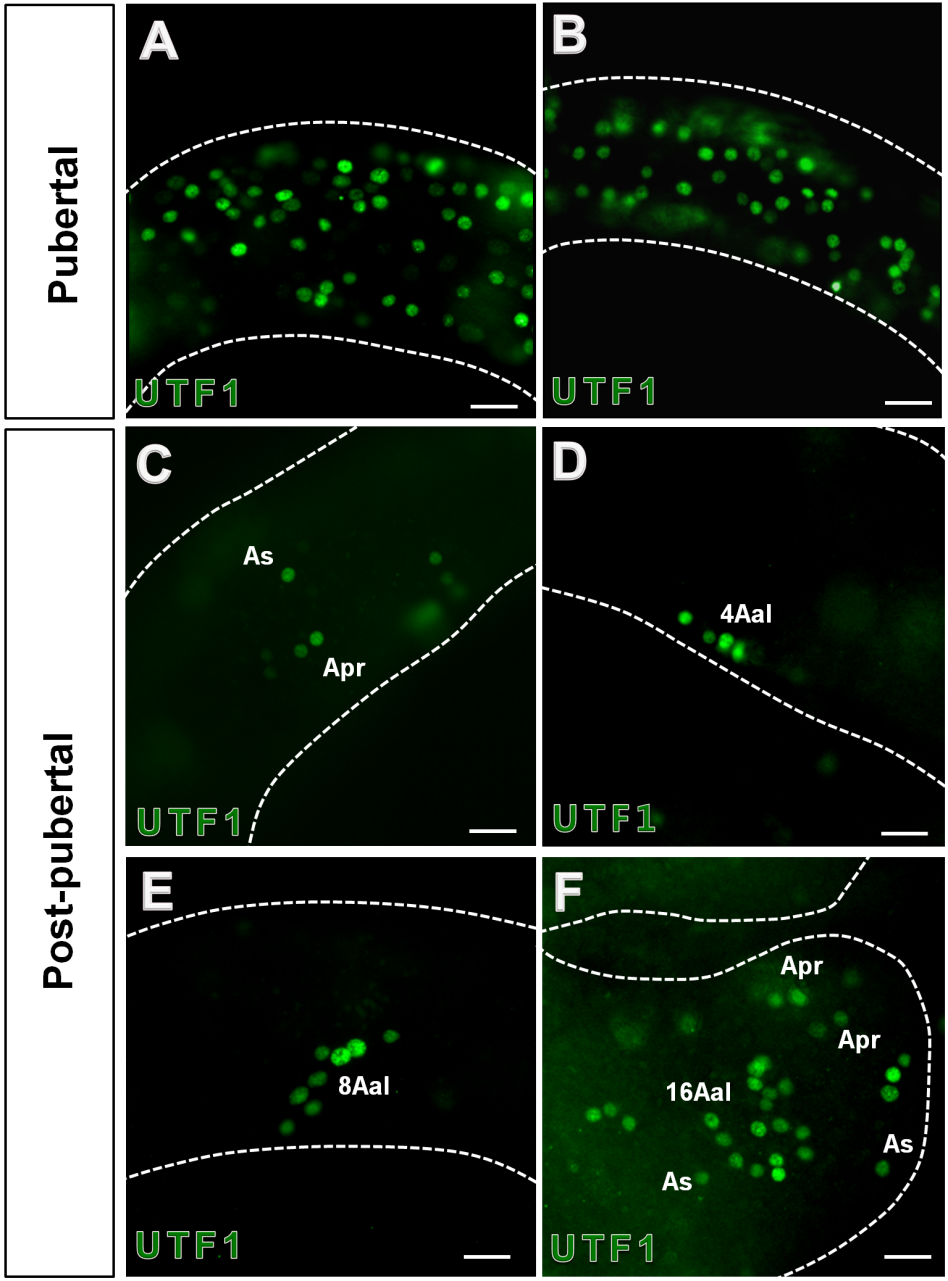
Stage-dependent clonal organization of UTF1-positive spermatogonia in stallion seminiferous tubules. Immunofluorescent staining for UTF1 was performed onto whole-mounted tubules. UTF-1 positive germ cells (green fluorescence) were observed in pubertal (A–B) and post-pubertal (C–F) stallion tubules. In pubertal stages, most UTF1 positive cells appeared to be A_s_ spermatogonia (A–B). In post-pubertal stages, A_s_, A_pr_, and chains of 4, 8, 16 A_al_ spermatogoina were found to be UTF1 positive. Bar = 50 µm.

To determine the size of the spermatogonia subpopulations expressing UTF1 in stallions, immunohistochemical analysis was performed on whole mount preparations of seminiferous tubules of pubertal and post-pubertal stallions. In the pubertal tubules, it was demanding to identify the size of UTF1 positive germ cell colony because of massive population of UTF1 germ cells within the tubules. However, most UTF1 positive germ cells in this stage appeared to be A_s_ spermatogonia (Figs. 3A and B). In the post-pubertal stallion tubules, whereas, A_s_, A_pr_, chains of 4, 8, and 16 A_al_ spermatogonia showed positive signals for UTF1 (Figs. 3C–F). No longer than chains of 16 A_al_ spermatogonia was observed in this study.

### 4. Absence of UTF1 expression in Sertoli cells of stallion testis

**Figure 4:**
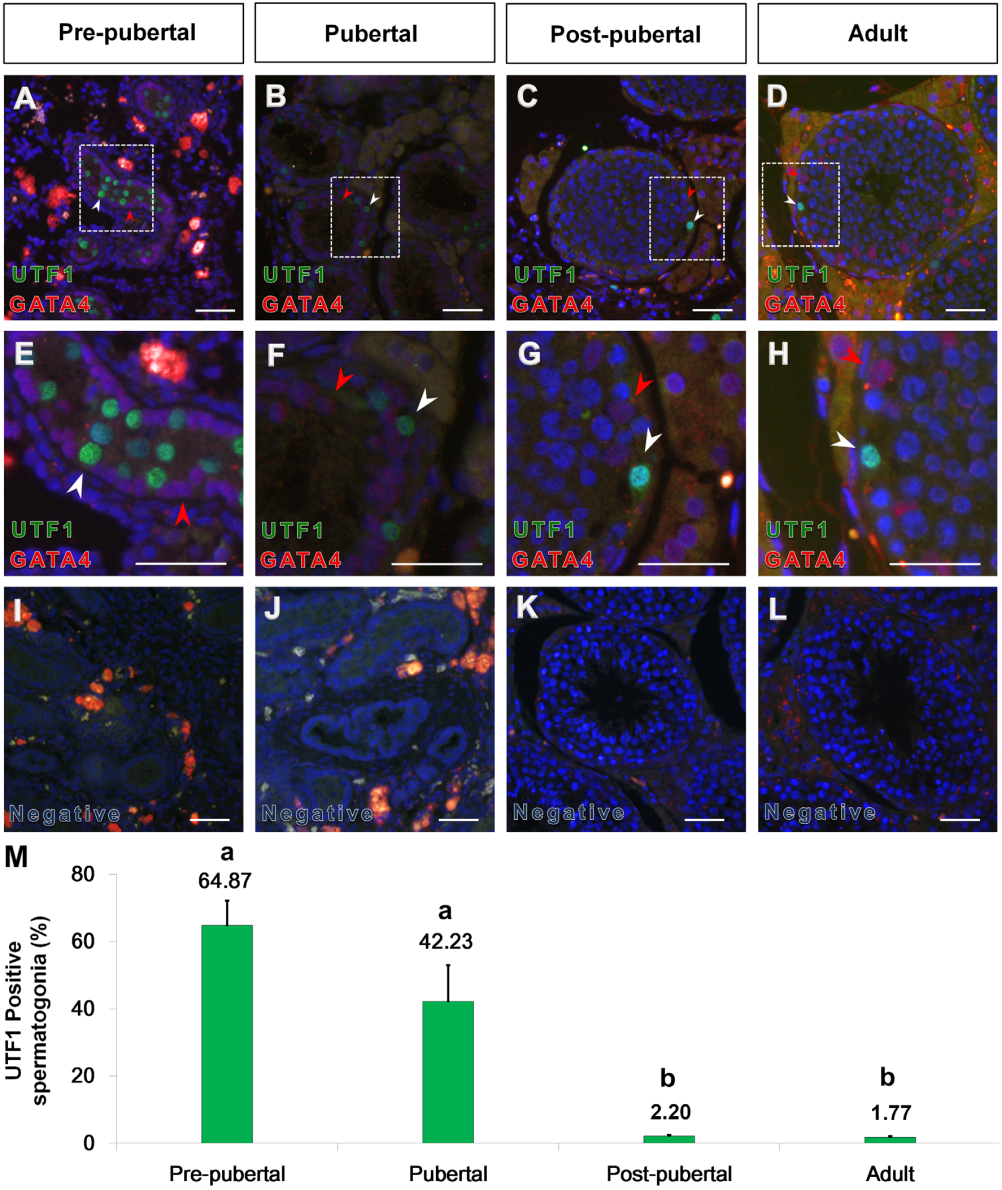
Stage-dependent co-localization of UTF1 and GATA4 in the seminiferous tubule cross-section of the stallion testes. Immunolabeling of UTF1 (green fluorescence) and GATA4 (red fluorescence) were observed in pre-pubertal (A and E), pubertal (B and F), post-pubertal (C and G), and adult (D and H) stallion testes. At the pre-pubertal stage, GATA4-positive cells were located close to the basement membrane of the seminiferous tubules. UTF1 was not co-immunolabeled with GATA4 (A and E). At the pubertal stage, UTF1 was localized between the Sertoli cells in the compartment of the basement membrane (B and F). At the post-pubertal and adult stages, completion of spermatogenesis and lumen opening were evident (C and D). UTF1-positive germ cells were observed in the basal compartment of seminiferous tubules. UTF1 immunolabeling was not observed in Leydig, Sertoli, or myoid cells at any reproductive stages. No immunolabeling was observed in the negative control tissues (I, J, K, and L). The area indicated by the broken line-white box (e.g., A, B, C, and D) is enlarged (e.g., E, F, G, and H). The white arrow head indicates spermatogonia immunolabeled with UTF1; the red arrow head indicates Sertoli cells immunolabeled with GATA4. Bar = 200 µm. The graph shows % of UTF1 stained spermatogonia out of randomly selected 2000 spermatogonia with error bars at different reproductive stages (M). Percentage with different superscripts indicate significant different (p<0.05).

Immunofluorescence was performed to determine the co-immunolabeling pattern of UTF1 and GATA4 (Fig. 4). GATA4 is widely used as a molecular marker for Sertoli cells [2]. In pre-pubertal tissues, GATA4-positive cells resided next to the basement membrane of the seminiferous tubules (Figs. 4A and E). UTF1-positive spermatogonia were not co-immunolabeled with GATA4 (Figs. 4A and E). At the pubertal stage, UTF1-positive cells were located between Sertoli cells in the compartment of basement membrane (Figs. 4B and F). At the post-pubertal and adult stages, a complete generation of spermatogenesis and lumen opening were evident (Figs. 4C and D). At these stages, UTF1 immunolabeling was observed in the nuclei of germ cells adjacent to the basement membrane of the seminiferous tubules, and these cells were juxtaposed to Sertoli cells (Figs. 4C, D, G, and H). No UTF1 immunolabeling was observed in Sertoli cells at any reproductive stage. The rate of UTF1 stained spermatogoina out of randomly selected 2000 spermatogonia was significantly lower at post-pubertal (2.2±0.25%, n = 3) and adult (1.77±0.32%, n = 3) compared to the ratio at pre-pubertal (64.87±7.36%, n = 3) and pubertal (42.23±10.77%, n = 3) (p<0.05, Fig. 4 M).

### 5. Stage-dependent co-localization of UTF1 and DAZL in stallion germ cells

**Figure 5:**
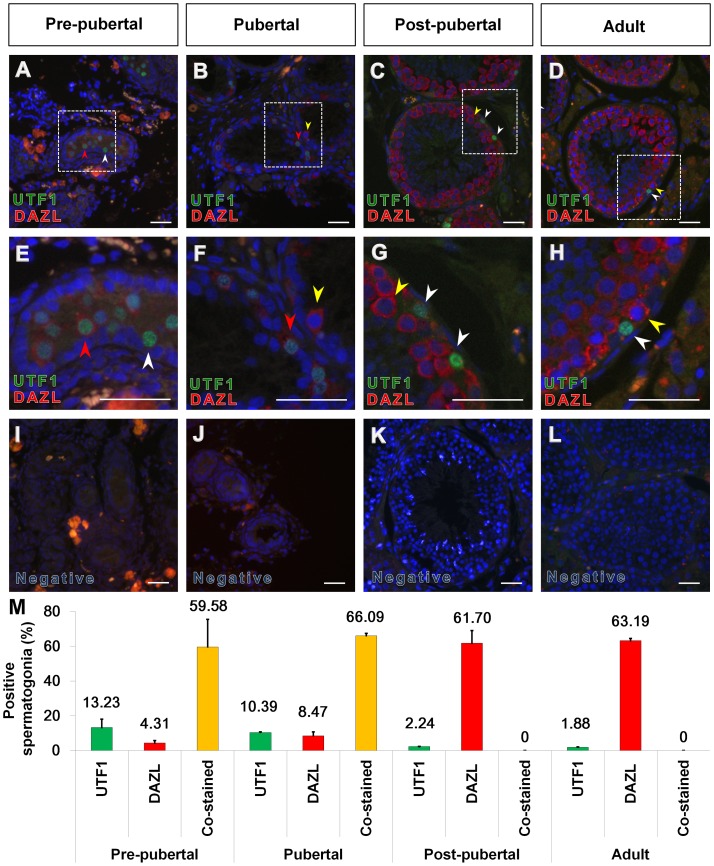
Stage-dependent co-localization of UTF1 and DAZL in the seminiferous tubule cross-section of stallion testes. Immunolabeling of UTF1 (green fluorescence) and DAZL (red fluorescence) were observed in pre-pubertal (A and E), pubertal (B and F), post-pubertal (C and G), and adult (D and H) stallion testes. At the pre-pubertal stage, most UTF1-positive spermatogonia were co-immunolabeled with DAZL (A and E, red arrow head), whereas germ cells stained with UTF1 only was also observed (A and E, white arrow head). At the pubertal stage, germ cells showed two types of expression patterns, which included germ cells expressing both UTF1 and DAZL (B and F, red arrow) and germ cells expressing DAZL only (yellow arrow). At the post-pubertal and adult stages, UTF1-positive germ cells were not co-immunolabeled with DAZL (C, D, G, H, white arrow). Insets show negative controls treated with normal rabbit serum in place of the primary antibody. No immunolabeling was noticed in the negative control (I, J, K, and L). The area indicated by a broken line-white box (e.g., A, B, C, D) is enlarged in the lower panel (e.g., E, F, G, H). The white arrow head indicates spermatogonia immunolabeled with UTF1, but not co-immunolabeled with DAZL. The red arrow head indicates co-immunolabeling of UTF1 with DAZL. The yellow arrow head indicates the cytoplasm of single germ cells with DAZL. Bar = 200 µm. The graph shows % of UTF1 and/or DAZL stained spermatogonia out of randomly selected 2000 spermatogonia with error bars at different reproductive stages (n = 3, M).

DAZL is a well-known germ cell marker of various species [17]–[20]. In our laboratory, we reported that most spermatogonia and primary spermatocytes were immunolabeled with DAZL protein at the post-pubertal and adult stages, although some spermatogonia did not show DAZL immunolabeling. To further investigate the subpopulations of DAZL-positive germ cells, co-immunolabeling of UTF1 with DAZL was conducted using testicular tissues of different reproductive stages (Fig. 5). At the pre-pubertal and pubertal stage, most UTF1-positive spermatogonia were co-stained with DAZL, although we also observed germ cells stained with UTF1 or DAZL only (Figs. 5A, B, E, F, and M). In the post-pubertal and adult stages, UTF1-positive germ cells were not co-stained with DAZL (Figs. 5C, D, G, H, and M). At these stages, spermatogonia were stained with UTF1 or DAZL only, and most spermatogonia were stained with DAZL only (Figs. 5C, D, G, H, and M). No immunolabeling was observed in the negative control (Figs. 5I–L).

### 6. Possible use of UTF1 antibody for immunocytochemistry

**Figure 6:**
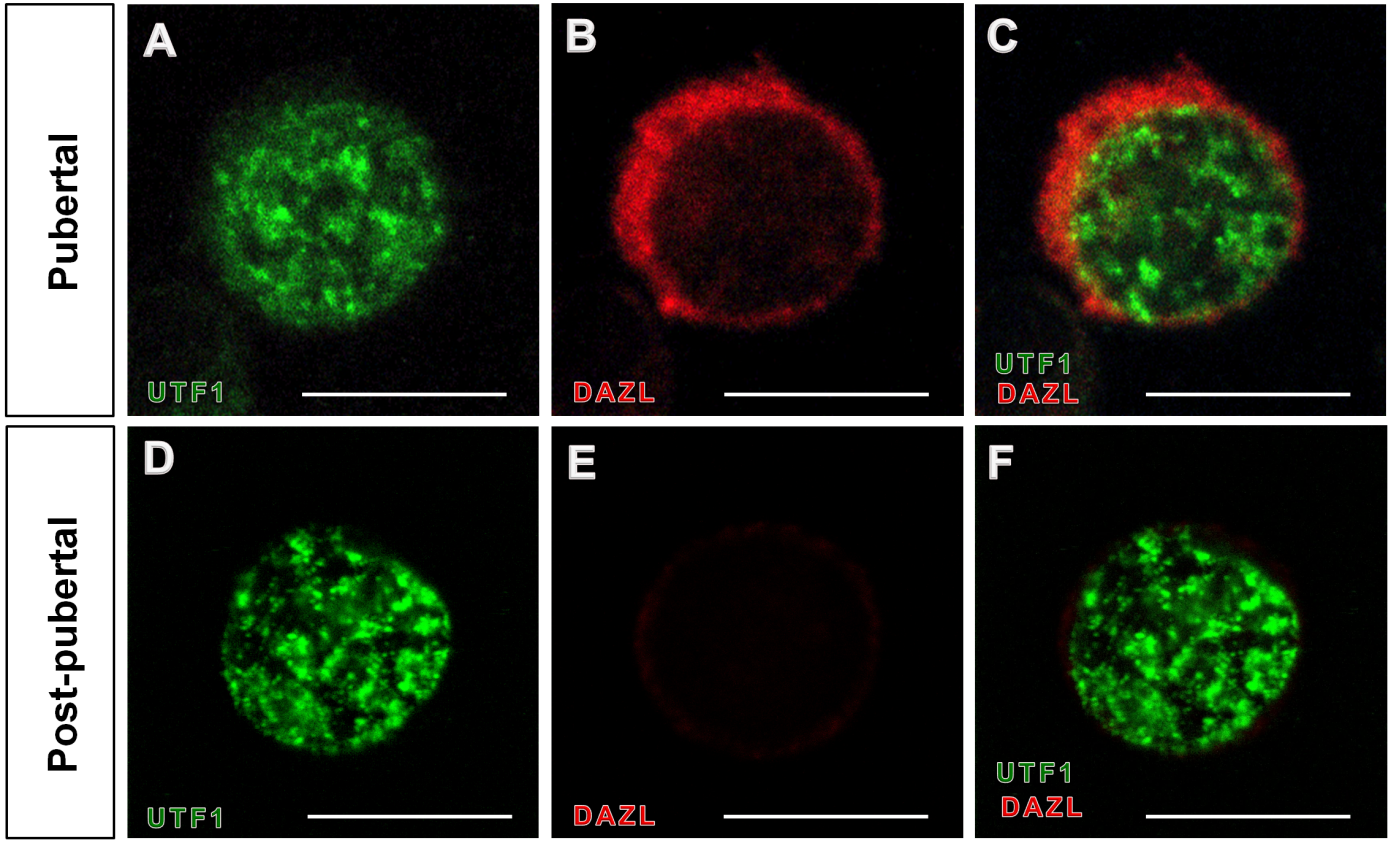
Immunocytochemistry with UTF1 and DAZL antibody in horse spermatogonia. The UTF1 and DAZL expression pattern in a single germ cell from pubertal and post-pubertal stallion testes were examined by immunocytochemistry (A–F). In pubertal stage, double-immunolabeling of UTF1 and DAZL were observed (A–C). In post-pubertal stage, germ cells were stained with UTF1 only (D–F). Bar = 30 µm.

We performed immunocytochemistry with UTF1 on germ cells to investigate possible use of the UTF1 antibody as a marker for undifferentiated spermatogonial stem cells in *in vitro* studies. To determine the optimal germ cell fixative for UTF1 immunocytochemistry, four different cell fixatives, including 1) ice-cold 100% MeOH, 2) 100% acetone, 3) 50% MeOH and 50% EtOH, and 4) 50% acetone and 50% EtOH were used to fix single germ cells onto slides. UTF1 immunolabeling of germ cell nuclei was successfully observed in germ cells fixed with all fixatives tested for this study. Co-immunostaining with both UTF1 and DAZL showed that the UTF1 protein was localized in nuclei of spermatogonia at pubertal and post-pubertal stallions, but DAZL staining was detected in the cytoplasm of undifferentiated spermatogonia at pubertal only (Fig. 6A–F).

## Discussion

Finding a putative marker for undifferentiated spermatogonia will contribute to the development of a new assistant reproductive technique using SSCs, as well as to better understanding the biological activity of spermatogonia for stallion sperm production. The present study was conducted to identify the subpopulations of spermatogonia that expressed UTF1 and to determine the changes in the pattern of UTF1 expression at different reproductive stages. Our immunohistological evidence demonstrated that UTF1-positive germ cells were present at every reproductive stage, and the localization of UTF1 was limited to the nuclei of germ cells. The pattern and localization of UTF1 in stallion testes was similar to that observed in humans [9] and rats [10], indicating that the pattern and localization of UTF1 expression are highly conserved in mammals. Several histological characteristics such as the location of germ cells in the seminiferous tubules and the shape of UTF1-positive cell membrane and nuclei were evaluated to identify UTF1-expressing subpopulations of spermatogonia. In post-pubertal and adult testes, UTF1-positive germ cells reside basally within the tubule. UTF1-positive cells located basally were flattened, whereas the other side of surface facing the other cells ranged from oval to round. These UTF1 immunofluorescence findings indicate that these cells are spermatogonia [21]. A recent study involving equine spermatogonia stem cells demonstrated that the population of Aund cells at each stage was approximately 200 cells per 1000 Sertoli cell nuclei [3]. In contrast, the population of A1, A2, A3, B1, or B2 spermatogonia was >200 cells per 1,000 Sertoli cell nuclei at each stage [3]. In this study, the number of UTF1-positive germ cell populations per 1,000 Sertoli cells in post-pubertal and adult stallions was less (78.42±17.83; n = 8) than Aund population previously reported in stallions [3]. The number of UTF1-positive germ cells per seminiferous tubule cross-section was 1.48±0.24 (n = 8), suggesting that UTF1 is expressed in early subpopulations of undifferentiated spermatogonia. Also immunofluorescent analysis of whole mounted tubules showed that UTF1-positive germ cells consisted of A_s_, A_pr_, and 4, 8, 16 chains of A_al_ spermatogonia. This result led us to conclude that UTF1 can be used as a marker for undifferentiated spermatogoina such as A_s_, A_pr_, and 4, 8, 16 chains of A_al_ spermatogonia.

The present study demonstrated that the ratio of UTF1-positive spermatogonia decreases during the transition from pubertal to post-pubertal. This finding was also reported in rats, in which the population of UTF1-positive cells in a cross-section decreased with age [10]. A possible explanation for this change is that UTF1-positive germ cells accumulate in the stunted seminiferous tubule at an early reproductive stage are distributed to the extended seminiferous tubule as the diameter, volume, and length of the single seminiferous tubule increase with active spermatogenesis after puberty [22].

DAZL is expressed in germ cells of mice [18], [20], rats [19], pigs [23], rhesus monkeys [15], bulls [24], and humans [17], [18], and its expression pattern is species- and developmental stage-dependent. Our research group has previously reported on the stage-dependent DAZL expression in stallion germ cells [12]. In the present study, germ cells co-stained with both UTF1 and DAZL were only observed at the pre-pubertal and pubertal stages, but not at the post-pubertal and adult stages, indicating that co-localization of UTF-1 and DAZL in the germ cells is stage-dependent [12]. DAZL is a widely known marker for differentiated spermatogonia [17], [19], [20]. Schrans-Stassen and coworkers also demonstrated that the differentiation of A_al_ spermatogonia into A_1_ spermatogonia in mice is blocked by the absence of the RNA-binding protein encoded by the DAZL gene [12], [25]. These previous evidences support that UTF1-positive and DAZL negative spermatogonia found in all reproductive stages is undifferentiated spermatogonia. In embryonic stem cells (ESCs), UTF1 expression is rapidly downregulated during ESC differentiation, indicating that UTF1 is a marker for undifferentiated ESCs and possibly plays an important role in maintain this status. Kooistra and coworkers suggested that the function of UTF1 is to maintain undifferentiated status by averting decondensation of chromatin and irregular gene expression of ESCs, which initiate the differentiation process [26]. These findings thus suggest that the UTF1-positive and DAZL-negative germ cells in post-pubertal and adult stallions are possibly undifferentiated SSCs and have the capacity to undergo self-renewal. This fact corresponds with the conclusion of an earlier study involving rats [10]. UTF1 induces rapid cell proliferation and promotes pluripotency of ESCs via coupling the core pluripotency factors with Myc and the PRC2 network [27], [28]. Based on the function of UTF1 in embryonic stem cells, we speculate that UTF1 might be involved in promoting self-renewal and maintaining the characteristics of undifferentiated spermatogonial stem cells. However, interestingly some UTF1 positive spermatogonia, but not all, were stained with DAZL in pre-pubertal and pubertal stages. We previously speculated that DAZL positive germ cells are differentiated spermatogonia in pre-pubertal and pubertal stage [12]. However, it is not clear whether these DAZL positive spermatogonia in early reproductive stages are differentiated or undifferentiated because some of DAZL positive spermatogonia are also stained with UTF1. Further study such as co-staining with another undifferentiated spermatogonia marker and DAZL in spermatogonia of pre-pubertal and pubertal stages is warranted to identify the status of the double stained spermatogonia at pre-pubertal and pubertal stage. The reproductive stage-dependent UTF1 and DAZL co-staining pattern also suggests that the status of UTF1 or DAZL positive spermatogonia may change with the reproductive stage of stallions.

The western blot result showed a protein band at 36 and 26 kDa. Despite of the presence of unexpected band at 26 kDa, UTF1 staining pattern in the spermatogonia using the antibody (ab3383) in this present study and another study in rats [10] is similar with the UTF1 staining pattern in the spermatogoina of other spices using different UTF1 antibody [9]. Thus, it appears that the band at 26 kDa is non-specific band.

In summary, the present study has demonstrated that UTF1 is a putative marker for early subpopulations of undifferentiated spermatogonial stem cells in stallion testes, and this marker can be applied to monitoring SSCs for the study of undifferentiated spermatogonial stem cells *in vitro*.
